# Therapeutic efficacy of canagliflozin against hepatocarcinogenesis induced by CDD/DEN/TAA in a rat model: regulation of AMPK/HIF-1α/YAP-1/TAZ signaling pathways

**DOI:** 10.1007/s00210-025-04098-8

**Published:** 2025-05-29

**Authors:** Hany M. Fayed, Gihan F. Asaad, Marawan A. Elbaset, Ola A. Sharaf, Sawsan S. Mahmoud, Mohamed M. Amin, Zeinab A. El-Gendy, Fatma A. Ibrahim, Sherein S. Abdelgayed, Rehab F. Abdel-Rahman

**Affiliations:** 1 Department of Pharmacology, Medical Research and Clinical Studies Institute, National Research Centre, Giza, Egypt; 2 Biochemistry Department, Biotechnology Research Institute, National Research Centre, Giza, Egypt; 3 Department of Pathobiology, College of Veterinary Medicine, Tuskegee University, Tuskegee, AL 36088 USA; 4 Department of Pathology, Faculty of Veterinary Medicine, Cairo University, Giza, 12211 Egypt

**Keywords:** Hepatocellular carcinoma, Canagliflozin, Diethyl nitrosamine, Thioacetamide

## Abstract

Hepatocellular carcinoma (HCC) is the most prevalent type of primary liver cancer. Many medications that had been used for a long period to treat the illness were eventually stopped due to negative effects or the development of drug resistance in HCC patients. Canagliflozin (CANA), a sodium-glucose cotransporter 2 (SGLT2) inhibitor, showed in vitro anti-carcinogenic efficacy against various cancer models’ other livers. The current study used rat models to examine canagliflozin’s therapeutic role against experimentally induced HCC. A total of 32 rats were divided into four groups, eight in each: negative control; HCC control: rats were fed a choline-deficient diet (CDD) and subjected to diethyl nitrosamine and thioacetamide (DEN/TAA) injections for 15 weeks; and treated groups: rats were given CANA (10 and 20 mg/kg b.wt.) orally from the 7th week of the experiment till the end. All the measured markers of HCC, liver function, and inflammatory markers were elevated in the HCC control group compared to the negative control (CTRL). Regarding immunohistochemistry, the HCC group showed downregulation in caspase-3 expression and upregulation in PCNA expression. On the other hand, canagliflozin-treated groups showed dose-dependent improvement in the measured parameters associated with HCC. Moreover, canagliflozin therapy ameliorates the histopathological alterations in HCC-induced rats. Taken together, CANA exhibits anti-HCC effects by activating AMP-activated protein kinase (AMPK) and suppressing the HIF-1α/YAP/TAZ pathway, making it a forthcoming therapeutic option for HCC.

## Introduction

Globally, hepatocellular carcinoma (HCC) is one of the main causes of cancer-related mortality (Elleithi et al. 2024). The World Health Organization (WHO) predicts that more than 1 million people would die from HCC worldwide each year by 2030. Approximately 800,000 new cases of primary liver cancer are discovered annually worldwide (Hassan et al. 2024). With 80–90% of all cases, HCC is the most prevalent form of primary liver cancer (Kim 2024). Hepatitis B and hepatitis C virus infections, alcohol consumption, metabolic liver illnesses including diabetes and non-alcoholic fatty liver disease (NAFLD), environmental contaminants, nitrosamine use, and obesity are all strongly associated with chronic liver inflammations, cirrhosis, and HCC development (Afifi et al. 2014; Abd El-Rahman and Fayed 2019). For patients with liver cancer, the prognosis is poor because of the late stage at diagnosis, resistance to current treatment, and high risk of recurrence. Five years following surgery, the recurrence rate is over 50%, and the overall survival rate for individuals with liver cancer is 18% (Sas et al. 2022). Currently, available chemotherapeutic medications are ineffective at controlling the growth of tumors, and drug resistance is widespread. To increase the overall survival rate of patients with HCC, new chemotherapeutic drugs are therefore desperately needed to block HCC at an early stage (Jo et al. 2016).

The precise processes that result in the development of liver oncogenesis are still unknown (Zhang and Zhou 2019). Here, we suggest a signaling strategy where the upstream regulator of Hippo signaling is the cellular energy level. The Hippo pathway has become a crucial tumor suppressor mechanism that influences the growth, regeneration, and carcinogenesis of liver tissue. Briefly, Hippo signaling’s main function is to inhibit its downstream transcriptional coactivators, transcription regulator 1 (TAZ), and Yes-associated protein (YAP), which control cell division and proliferation (Johnson and Halder 2014; Patel et al. 2017). YAP or TAZ, a transcriptional co-activator, has been abnormally activated in a number of human malignancies, including HCC (Zhang and Zhou 2019). Energy stress has been shown in earlier research to limit YAP transcriptional activity and YAP-dependent transformation while inducing YAP cytoplasmic retention. AMP-activated protein kinase (AMPK), the primary metabolic sensor, and the upstream Hippo pathway elements Lats1/Lats2 and angiomotin-like 1 (AMOTL1) are necessary for these effects (DeRan et al. 2014). Since cellular energy and metabolites are necessary for survival and growth, AMPK activation inhibits YAP. In fact, AMPK is a negative regulator of YAP activity. AMPK activation promotes YAP cytoplasm localization, increases YAP phosphorylation, and inhibits YAP target genes (Wang et al. 2015). Furthermore, AMPK and drug resistance are tightly connected, according to several studies (Qin et al. 2019; Zhou et al. 2019). Certain natural products contain bioactive chemicals and tiny molecular inhibitors that activate AMPK, enhancing multidrug resistance (Wang et al. 2016). AMPK activators and chemotherapeutics work well together to limit tumor growth and extend the remission of malignancies of the breast, pancreas, prostate, lung, and ovaries (Wu et al. 2014; Duan et al. 2017). For this reason, AMPK/YAP/TAZ has become a valuable target for cancer treatments.

Cancer models have been shown to overexpress sodium-glucose cotransporter 2 (SGLT2), a critical glucose transporter, which is accompanied by increased glucose uptake in both humans and mice. In vitro and in vivo tumor development can be effectively inhibited by blocking its expression (Basak et al. 2023). Novel SGLT2 inhibitor canagliflozin (CANA) has been shown to decrease patients’ glycemic levels by lowering renal glucose reabsorption (Chao 2014). Notably, in addition to controlling blood sugar levels, pre-clinical research revealed that CANA slowed the progression of lung, pancreatic, and prostate cancer (Villani et al. 2016). By inhibiting the progression of non-alcoholic steatohepatitis (NASH) and inducing apoptosis in HepG2 cells through caspase-3 activation, canagliflozin demonstrated anti-steatotic and anti-inflammatory properties (Jojima et al. 2019). A previous study showed that CANA re-sensitized HCC to cisplatin by triggering ferroptosis through dual effects on glycolysis and glutamine metabolism using cell culture and an in vivo mouse tumor model (Zeng et al. 2023). The effectiveness of CANA against HCC was also investigated by Luo et al. using cell culture and in vivo mouse tumor model. By preventing the accumulation of HIF-1α protein, most likely by targeting the AKT/mTOR pathway, CANA reduced metastasis, angiogenesis, and metabolic reprogramming in HCC (Luo et al. 2021). Simultaneously, Hung et al.’s study, utilizing cell culture and an in vivo mouse tumor model, demonstrated that CANA blocked β-catenin, which could offer new ideas for treating HCC patients who also have diabetes (Hung et al. 2019). Despite the fact that the previously mentioned studies showed that CANA is effective against HCC, no research has examined its effectiveness against HCC caused by CDD/DEN/TAA, which is similar to the course of HCC in humans that results from damage, fibrosis, cirrhosis, and cancer.

Therefore, we conducted this research to assess the potential therapeutic benefit of canagliflozin on CDD/DEN/TAA-induced HCC in rats and explore the role of AMPK/YAP/TAZ pathways that are known to mediate the advancement of liver cancer.

## Materials and methods

### Experimental animals

One-month-old weaned Wistar rats (50 and 80 g) selected for this investigation were procured from the animal house colony of the National Research Centre (NRC), Giza, Egypt. Rats were kept in cages with enough illumination, a humidity level of 60%, adequate ventilation, and a temperature of 25 ± 0.5 °C. Animals of all groups except the negative control were maintained in clean cages and given a choline-deficient diet (CDD) consisting of 60% corn, 21% soy protein (46%), 15% margarine, and 4% sucrose mix for 4 weeks. This was followed by a standard pellet diet until the end of the experiment, with water provided ad libitum. Experiments using animals were conducted according to the guidelines of the Medical Research Ethics Committee (MREC) at NRC, approval number 13060173.

### Drugs and chemicals

Sigma-Aldrich in Germany provided the diethyl nitrosamine (DEN) and thioacetamide (TAA). Tablets of canagliflozin (Invokana; 300 mg) were acquired from “Nile Pharmaceutical Company in Egypt.”

### Hepatocarcinogenesis model

**Fig. 1:**
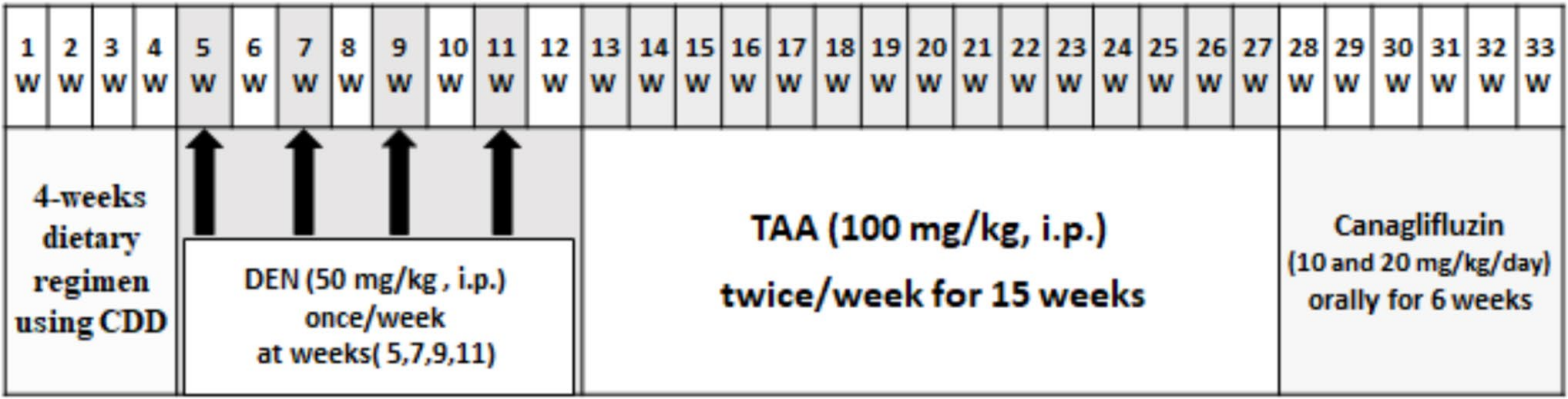
An illustration of the experimental design

Figure 1 summarizes the experimental time line. One-month-old weaned male rats weighing between 50 and 80 g were utilized in the experiment. Rats of the negative control group were kept on standard rat chow all over the experimental period. However, rats of the other groups were accessible to a choline-deficient diet for a duration of 4 weeks, followed by a standard diet until the end of the experiment (Newberne et al. 1982). Following a 4-week dietary regimen, rats received four intraperitoneal (i.p.) diethyl nitrosamine (DEN) 50 mg/kg injections over 8 weeks; each injection was 15 days apart (four strategic intervals) (Kurma et al. 2021). Subsequently, the animals received intraperitoneal injections of thioacetamide (TAA) at a dosage of 100 mg/kg, administered twice weekly for 15 weeks (Ayoub et al. 2022). Treatment of rats was continued for 6 successive weeks. A gross examination of the liver was done at the end of the experimentation.

### Design of experiment

Thirty-two rats were randomly allocated into four groups (8 rats each):

- Group I: The negative control rats (CTRL) received saline i.p. all over the experimental period.
- Group II: served as (HCC group).
- Group III and IV: the rats were orally administered canagliflozin at doses of (10 and 20 mg/kg/day, orally) (Mabrouk Gabr and Hassan 2020) for 6 weeks after induction of hepatocarcinogenesis.

### Sacrification and biological sample collection

Blood samples were taken from the tail vein of rat under light phenobarbital anesthesia. Centrifugation was utilized to separate the sera, which were then used for the biochemical analysis. The liver was extracted, saline-washed, blot-dried, and divided into three pieces. One slice was then submerged in 10% buffered formalin for immunohistochemical and histological examination. In 10% w/v phosphate-buffered saline, another slice was homogenized, and the supernatant was used for the ELISA test and the assessment of oxidative stress biomarkers. The remainder was frozen for PCR analysis at − 80 °C.

### Liver injury indicators

By utilizing enzymatic colorimetric techniques, the liver enzymes alanine aminotransferase (ALT) and aspartate aminotransferase (AST) were determined using Biodiagnostic kits, Egypt, Catalog No AL 10 31 (45) and AS 10 61 (45), respectively. Gamma-glutamyl transferase (GGT) level “(Cat# EZ009LQ)” was determined colorimetrically using kits manufactured by Biodiagnostic kits, Giza, Egypt.

### Analyzing the liver’s oxidative stress and antioxidant levels

Following the manufacturer’s instructions, kits from “Bio-diagnostic, Egypt” were used to evaluate the liver levels of “reduced glutathione (GSH), superoxide dismutase (SOD), and malondialdehyde (MDA)” (Cat# GR2511, SD2521, MD2529).

### Inflammatory indicators

STAT3, liver signal transducer, and activator of transcription 3 (Cat# SL1672Ra) and p-STAT-3 (Cat# SLD1757Ra) levels were determined with the ELISA technique using Sunlong Biotech Co., Ltd., China kits.

### HCC markers

Serum alpha-fetoprotein (AFP) (Cat# SL0042Ra), alpha-l-fucosidase (AFU) (Cat# SL0044Ra), carcinoembryonic antigen (CEA) (Cat# SL1244Ra), and transforming growth factor β1 (TGF-β1) (Cat# SL0705Ra) were determined by the Elisa technique using Sunlong Biotech Co., Ltd., China kits.

### Measurement of SIRT1, Nrf2, AMPK, and p-AMPK levels

Following the manufacturer’s instructions and using Sunlong Biotech Co., Ltd., China kits, the levels of “Nrf2 (NF-E2-related factor 2) (Cat# SL0985Ra), AMPK (AMP-activated protein kinase) (Cat# SL1695Ra), SIRT1 (Sirtuin 1) (Cat# SL1254Ra), and p-AMPK (Cat# SL0570Ra)” in liver tissues were assessed using the ELISA technique.

### Measurement of YAP-1, TAZ, and HIF-1α levels

YAP-1 (Cat# SL1731Ra), TAZ (China; Cat# SLD1758Ra), and HIF-1α (China; Cat# SL0357Ra) were assayed using Sunlong Biotech Co., Ltd., China kits.

### Quantitative real-time PCR to measure “AMPK and HIF-1α” gene expression

After 30 mg of tissue sample was added to 600 µl of RLT buffer with 10 µl β mercaptoethanol per 1 ml, the “QIAamp RNeasy Mini kit (Qiagen, Germany, GmbH)” was used to extract RNA from the tissue samples. Adapter sets that are connected to the “Qiagen tissue Lyser clamps” were used to insert the tubes and homogenize the samples. A high-speed (30 Hz) shaking step was used for 2 min in order to cause disruption. To purify total RNA from animal tissues, 70% ethanol was added to the cleaned lysate using the “QIAamp RNeasy Mini kit (Qiagen, Germany, GmbH)” method. Note: DNase digestion was performed on the column to eliminate any leftover DNA. “Metabion (Germany)” supplied the oligonucleotide primers, which are listed in Table 1. The 25 µl reaction contained 12.5 µl of the 2 × QuantiTect SYBR Green PCR Master Mix (Qiagen, Germany, GmbH), 0.25 µl of RevertAid Reverse Transcriptase (200 U/µL) (Thermo Fisher), 0.5 µl of each primer at a concentration of 20 pmol, 8.25 µl of water, and 3 µl of RNA template. To perform the reaction, a “Stratagene MX3005P real-time PCR device” was used. Utilizing the “StratageneMX3005P program,” the amplification curves and ct values were acquired. The variance in gene expression on the RNA of the different samples was evaluated by comparing each sample’s CT with that of the positive control group using the following ratio: 2^-ΔΔct^, in line with Yuan et al. (Yuan et al. 2006) “ΔΔCt” technique. Nevertheless, “ΔCt reference − ΔCt target” equals ΔΔCt.

$$''\triangle\mathrm{Ct}\;\mathrm{target}=\mathrm{Ct}\;\mathrm{control}-\mathrm{Ct}\;\mathrm{treatment}\;\mathrm{and}\;\triangle\mathrm{Ct}\;\mathrm{reference}=\mathrm{Ct}\;\mathrm{control}-\mathrm{Ct}\;\mathrm{treatment}.''$$

**Table 1:** A list of primers for qPCR

Target genes		Primers sequences
*AMPK* (McCrimmon et al. 2006)	F	ATCCGCAGAGAGATCCAGAA
	R	CGTCGACTCTCCTTTTCGTC
HIF-1α (He et al. 2014)	F	GGACGATGAACATCAAGTCAGCA
	R	GGAATGGGTTCACAAATCAGCAC
Rat ß. Actin (Banni et al. 2010)	F	TCCTCCTGAGCGCAAGTACTCT
	R	GCTCAGTAACAGTCCGCCTAGAA

### Histopathological analysis

Livers were routinely processed after being taken from the various experimental groups. Hematoxylin and Eosin (H&E) (Bancroft and Gamble 2008) were used to stain the 5-micron-thick paraffin-embedded blocks so they could be examined histopathologically using “a light microscope (Olympus BX50, Japan).”

### Classification of histopathological alterations

The following criteria were used to grade the histological changes in the livers: (0) indicated no changes, and (+ +), (+ + +), and (+ + + +) indicated mild, moderate, and severe changes appropriately (Arsad et al. 2014).

### Immunohistochemical analysis

After cutting liver tissue sections onto sticky slides, deparaffinizing them, and rehydrating them with distilled water, a heat-induced epitope retrieval step was carried out. The tissue sections were then incubated for an hour at room temperature with “primary anti-caspase-3 (at a dilution of 1:200, sc-7272; Santa Cruz Biotechnology, Inc., Heidelberg, Germany) and anti-PCNA (at a dilution of 1:1000, 10,205–2-AP; Proteintech, Germany).” In accordance with the manufacturer’s instructions, the color was developed after washing using an HRP-labeled detection kit (BioSB, USA). Myer hematoxylin was used as a counterstain. By avoiding incubation with primary antibodies, negative control slides were acquired. The mean area % for each group was used to quantify positive expressiveness.

### Statistical approach

Before the statistical analysis, data values were checked for normality using the Shapiro test, and heteroscedasticity was checked using the Brown-Forsythe test. The data were presented as means ± SEM. Data were processed by one-way ANOVA followed by the Tukey as a post-hoc test. In contrast, non-parametric values were presented as median ± interquartile range and analyzed by the Kruskal–Wallis test followed by Dunn’s test; additionally, the exact* p*-value between each compared pairwise group is stated above the pairwise bar. GraphPad Prism software (version 10, San Diego, CA, USA) was utilized to establish the represented graphs. The significance level was set to be *p* ≤ 0.05 for all statistical tests.

## Results

### Liver enzyme activity: ALT, AST, and GGT levels

**Fig. 2:**
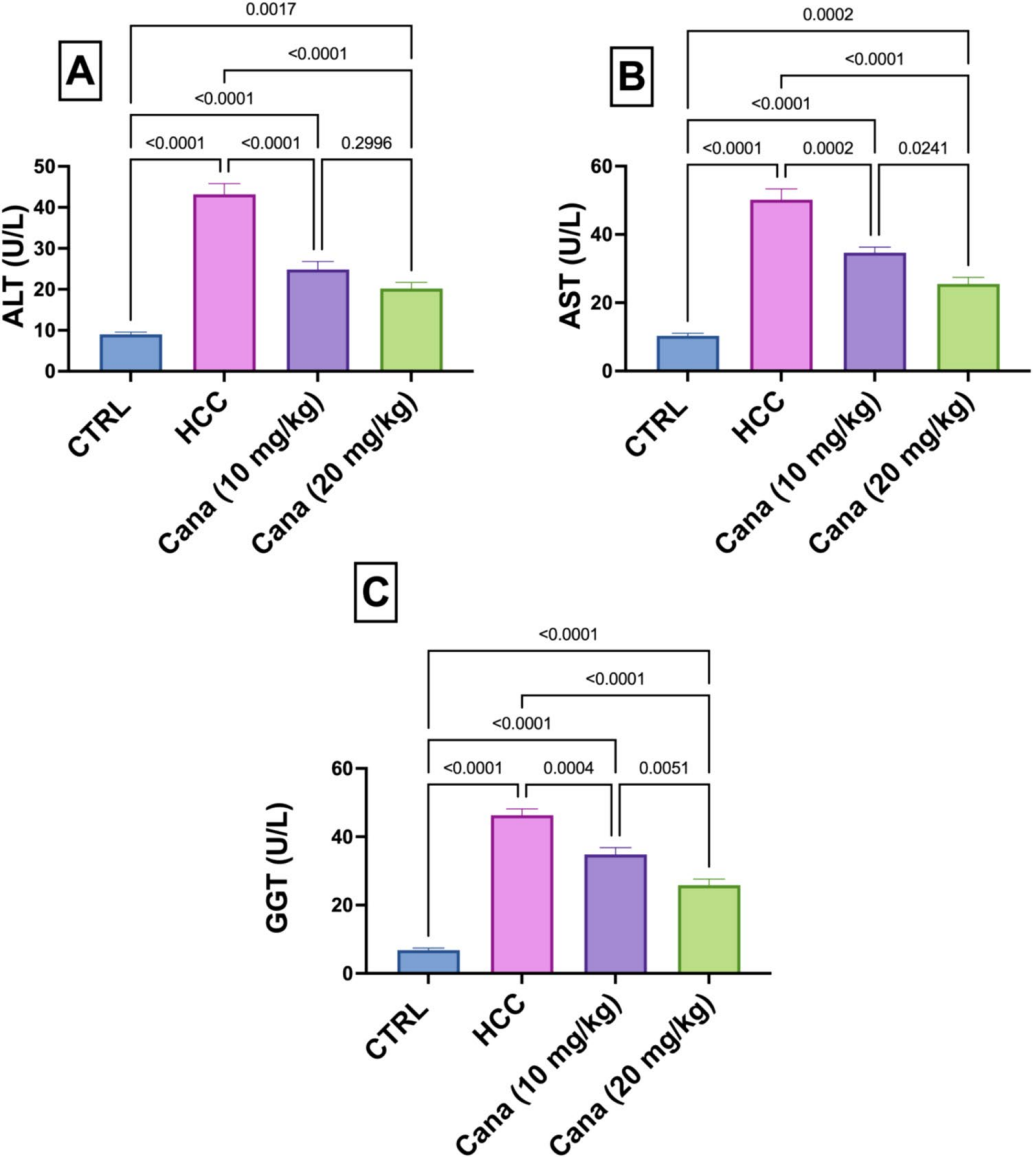
Assessment of canagliflozin effect on liver function: “ALT, AST, and GGT” in HCC rats. **A** “Serum ALT” (U/L). **B** “Serum AST” (U/L). **C** “Serum GGT” (U/L). Data are presented as mean ± SEM. The exact* p*-value between each compared pairwise group is stated above the pairwise bar. HCC, hepatocellular carcinoma; Cana, canagliflozin

In the HCC group, ALT, AST, and GGT levels were significantly elevated compared to the control group, indicating severe liver injury. ALT levels in the HCC group increased to 479.7% of the control group (*p* ≤ 0.05). Treatment with canagliflozin (Cana) at 10 mg/kg and 20 mg/kg reduced ALT levels to 57.5% and 46.7% of the HCC group, respectively, with the high dose showing a more significant reduction (*p* ≤ 0.05). AST levels in the HCC group rose to 485.6% of the control group (*p* ≤ 0.05), while treatment with Cana at 10 mg/kg and 20 mg/kg reduced AST levels to 69.1% and 50.8% of the HCC group, respectively (*p* ≤ 0.05). Similarly, GGT levels in the HCC group increased to 678.3% of the control group (*p* ≤ 0.05). Treatment with Cana at 10 mg/kg and 20 mg/kg reduced GGT levels to 75.2% and 55.8% of the HCC group, respectively (*p* ≤ 0.05). Despite significant improvements, none of the treated groups fully normalized to control levels (Fig. 2).

### Oxidative stress and antioxidant markers: MDA, SOD, and GSH levels

**Fig. 3:**
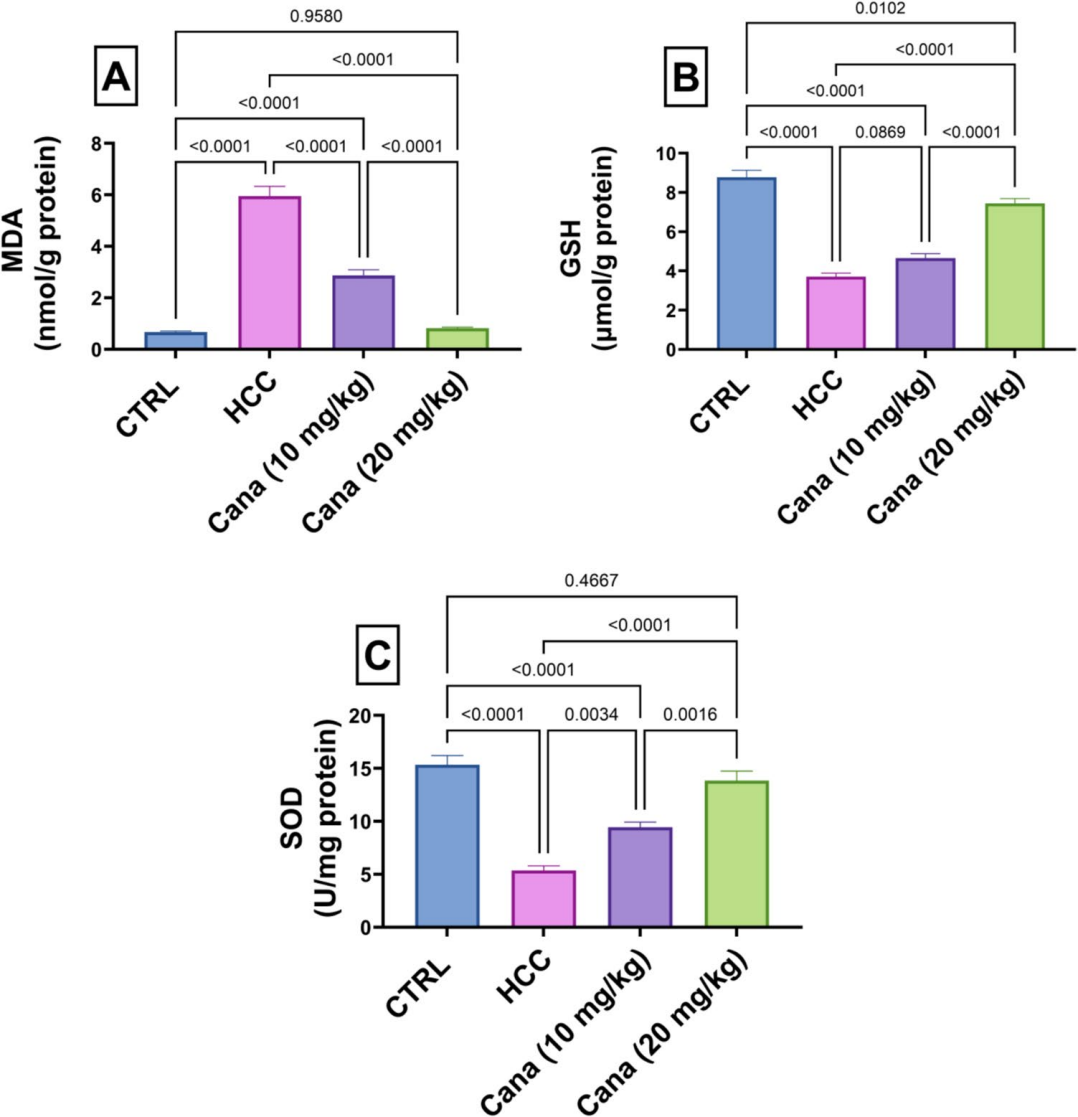
Assessment of canagliflozin effect on oxidative stress markers: “MDA, GSH, and SOD” in HCC rats. **A** “Liver MDA” (nmol/g protein). **B** “Liver GSH” (µmol/g protein). **C** “Liver SOD” (U/mg protein) activity. Data are presented as mean ± SE. The exact *p*-value between each compared pairwise group is stated above the pairwise bar. HCC, hepatocellular carcinoma; Cana, canagliflozin

Liver MDA levels, a marker of oxidative stress, were significantly elevated in the HCC group, reaching 890.4% of the control group (*p* ≤ 0.05). Treatment with Cana at 10 mg/kg and 20 mg/kg reduced MDA levels to 48.2% and 13.9% of the HCC group, respectively, with the high dose showing near normalization to control levels (*p* ≤ 0.05). SOD activity, an antioxidant enzyme, was significantly reduced in the HCC group to 34.9% of the control group (*p* ≤ 0.05). Treatment with Cana at 10 mg/kg and 20 mg/kg increased SOD activity to 176.3% and 258.5% of the HCC group, respectively, with the high dose restoring levels close to normal (*p* ≤ 0.05). Similarly, GSH levels in the HCC group were reduced to 42.2% of the control group (*p* ≤ 0.05). Treatment with Cana at 10 mg/kg and 20 mg/kg increased GSH levels to 125.7% and 200.9% of the HCC group, respectively, with the high dose showing significant improvement (*p* ≤ 0.05) (Fig. 3).

### Tumor markers: AFP, AFU, and CEA levels

**Fig. 4:**
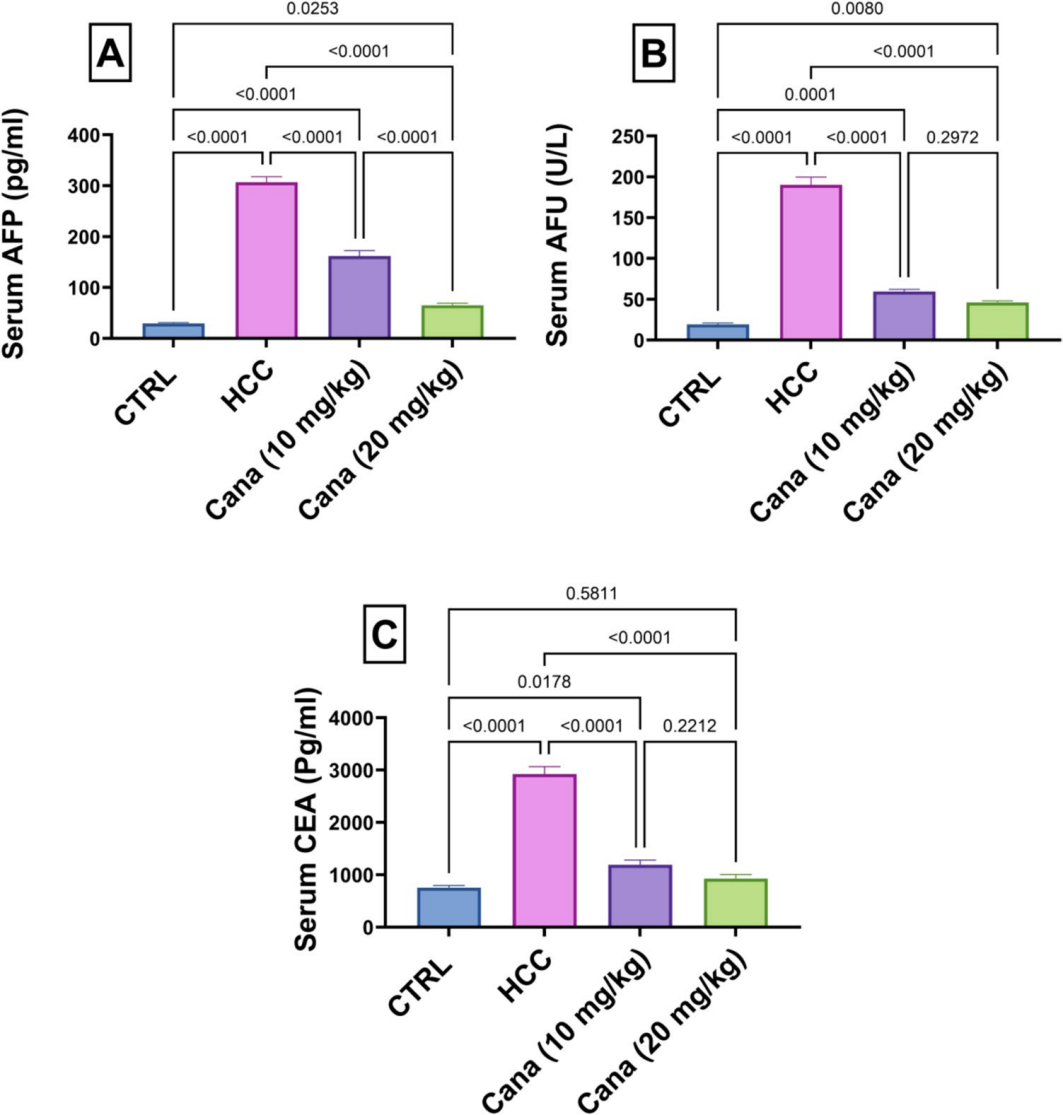
Assessment of canagliflozin effect on HCC markers: AFP, AFU, and CEA in HCC rats. **A** AFP (pg/ml). **B** AFU (U/L). **C** CEA (Pg/ml). Data are presented as mean ± SEM. The exact *p*-value between each compared pairwise group is stated above the pairwise bar. HCC, hepatocellular carcinoma; Cana, canagliflozin

Serum AFP, AFU, and CEA levels were significantly elevated in the HCC group, reflecting tumor progression. AFP levels in the HCC group increased to 1051.8% of the control group (*p* ≤ 0.05). Treatment with Cana at 10 mg/kg and 20 mg/kg reduced AFP levels to 52.7% and 21.2% of the HCC group, respectively, with the high dose showing a more significant reduction (*p* ≤ 0.05). AFU levels in the HCC group rose to 983.1% of the control group (*p* ≤ 0.05). Treatment with Cana at 10 mg/kg and 20 mg/kg reduced AFU levels to 31.3% and 24.3% of the HCC group, respectively (*p* ≤ 0.05). Similarly, CEA levels in the HCC group increased to 388.3% of the control group (*p* ≤ 0.05). Treatment with Cana at 10 mg/kg and 20 mg/kg reduced CEA levels to 40.8% and 31.6% of the HCC group, respectively (*p* ≤ 0.05). None of the treated groups fully normalized to control levels (Fig. 4).

### Hypoxia and fibrosis markers: YAP1, TAZ, and TGF-β1

**Fig. 5:**
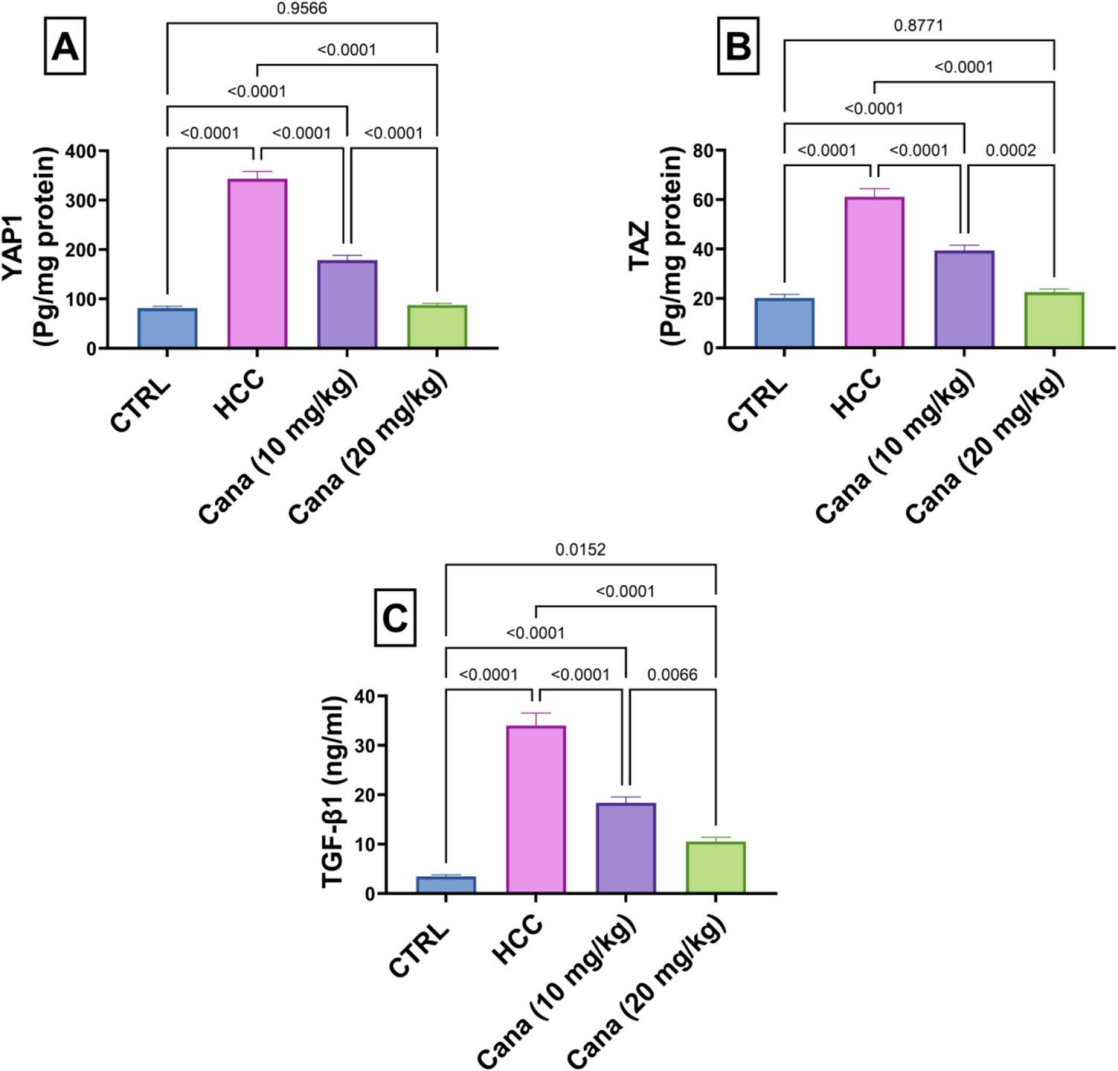
Assessment of canagliflozin effect on YAP1, TAZ, and TGF-β1 levels in HCC rats. **A** YAP-1 (Pg/mg protein). **B** TAZ (Pg/mg protein). **C** TGF-β1 (ng/ml). Data are presented as mean ± SEM. The exact *p*-value between each compared pairwise group is stated above the pairwise bar. HCC, hepatocellular carcinoma; Cana, canagliflozin

YAP1 levels in the HCC group increased to 423.1% of the control group (*p* ≤ 0.05). Treatment with Cana at 10 mg/kg and 20 mg/kg reduced YAP1 levels to 51.9% and 25.6% of the HCC group, respectively (*p* ≤ 0.05). TAZ levels in the HCC group increased to 303.2% of the control group (*p* ≤ 0.05). Treatment with Cana at 10 mg/kg and 20 mg/kg reduced TAZ levels to 64.3% and 36.8% of the HCC group, respectively (*p* ≤ 0.05). TGF-β1 levels in the HCC group increased to 985.5% of the control group (*p* ≤ 0.05). Treatment with Cana at 10 mg/kg and 20 mg/kg reduced TGF-β1 levels to 53.9% and 30.9% of the HCC group, respectively (*p* ≤ 0.05) (Fig. 5).

### Energy metabolism markers: AMPK, P-AMPK, AMPK mRNA relative expression, and P-AMPK/AMPK ratio

**Fig. 6:**
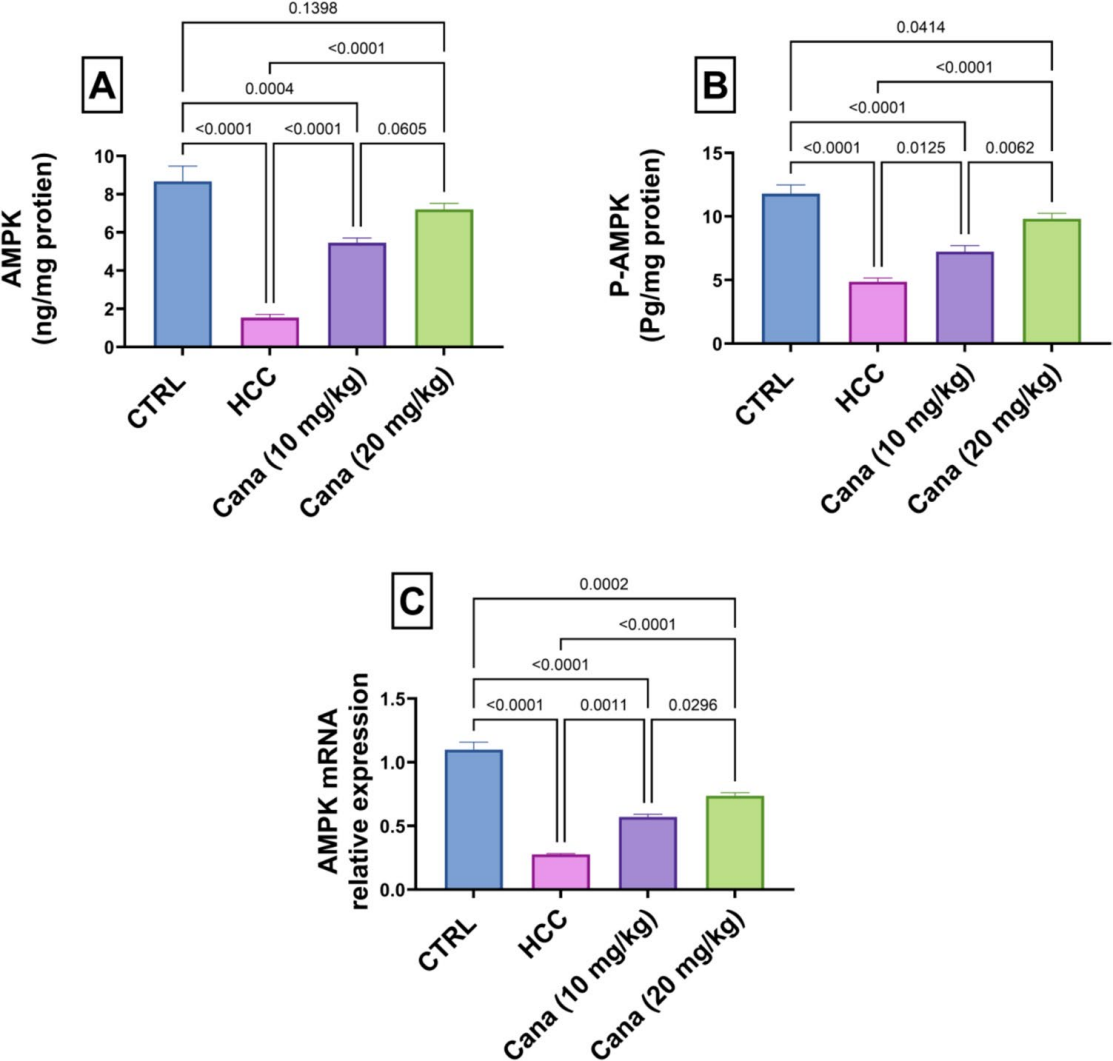
Assessment of canagliflozin effect on AMPK and p-AMPK levels in HCC rats. **A** AMPK (ng/mg protein). **B** p-AMPK (Pg/mg protein) and **C** AMPK mRNA relative expression. Data are presented as mean ± SEM. The exact *p*-value between each compared pairwise group is stated above the pairwise bar. HCC, hepatocellular carcinoma; Cana, canagliflozin

Liver AMPK levels in the HCC group were significantly reduced to 17.7% of the control group (*p* ≤ 0.05). Treatment with Cana at 10 mg/kg and 20 mg/kg increased AMPK levels to 355.5% and 480.0% of the HCC group, respectively (*p* ≤ 0.05). P-AMPK levels in the HCC group were reduced to 41.2% of the control group (*p* ≤ 0.05). Treatment with Cana at 10 mg/kg and 20 mg/kg increased P-AMPK levels to 148.6% and 201.7% of the HCC group, respectively (*p* ≤ 0.05). The P-AMPK/AMPK ratio in the HCC group was 2.7, compared to 1.36 in the control group. Treatment with Cana at 10 mg/kg and 20 mg/kg restored the ratio to 1.33 and 1.36, respectively, indicating normalization of energy metabolism (*p* ≤ 0.05) (Fig. 6).

In the HCC group, AMPK mRNA relative expression and liver AMPK protein levels were significantly reduced compared to the control group, while HIF-1α mRNA relative expression and liver HIF-1α protein levels were markedly elevated, indicating disrupted energy metabolism and increased hypoxia signaling. AMPK mRNA expression in the HCC group decreased to 25.2% of the control group (*p* ≤ 0.05). Treatment with canagliflozin (Cana) at 10 mg/kg and 20 mg/kg increased AMPK mRNA expression to 51.9% and 67.0% of the control group, respectively, with the high dose showing a more significant improvement (*p* ≤ 0.05) (Fig. 6).

### Antioxidant and stress response markers: SIRT1 and Nrf2

**Fig. 7:**
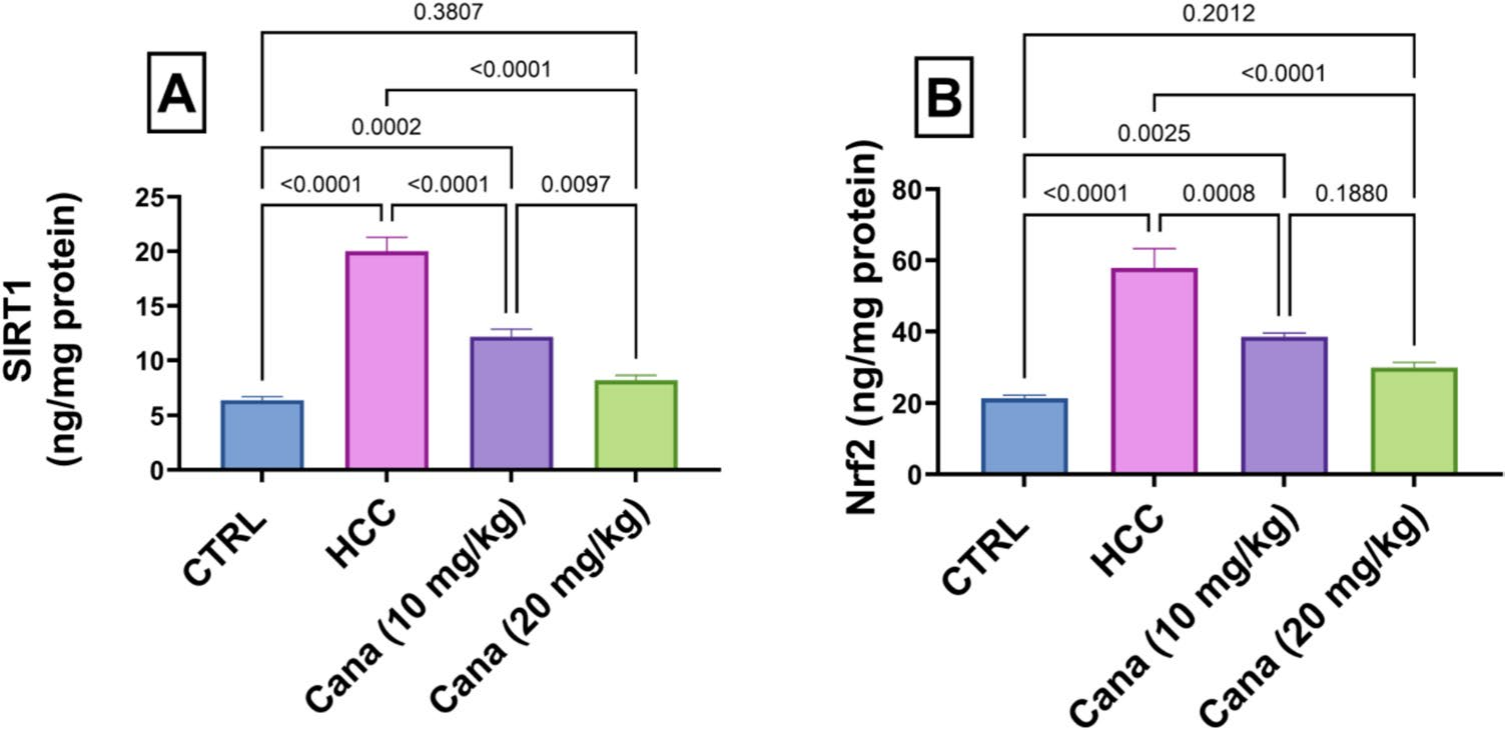
Assessment of canagliflozin effect on SIRT1 and Nrf2 levels in HCC rats. **A** SIRT1 (ng/mg protein). **B** Nrf2 (Pg/mg protein). Data are presented as mean ± SEM. The exact *p*-value between each compared pairwise group is stated above the pairwise bar. HCC, hepatocellular carcinoma; Cana, canagliflozin

Liver SIRT1 levels in the HCC group increased to 312.5% of the control group (*p* ≤ 0.05). Treatment with Cana at 10 mg/kg and 20 mg/kg reduced SIRT1 levels to 60.9% and 41.1% of the HCC group, respectively (*p* ≤ 0.05). Nrf2 levels in the HCC group increased to 271.2% of the control group (*p* ≤ 0.05). Treatment with Cana at 10 mg/kg and 20 mg/kg reduced Nrf2 levels to 66.6% and 51.6% of the HCC group, respectively (*p* ≤ 0.05). Despite significant improvements, neither marker fully normalized to control levels (Fig. 7).

### Inflammatory markers: STAT3, P-STAT3, and P-STAT3/STAT3 ratio

**Fig. 8:**
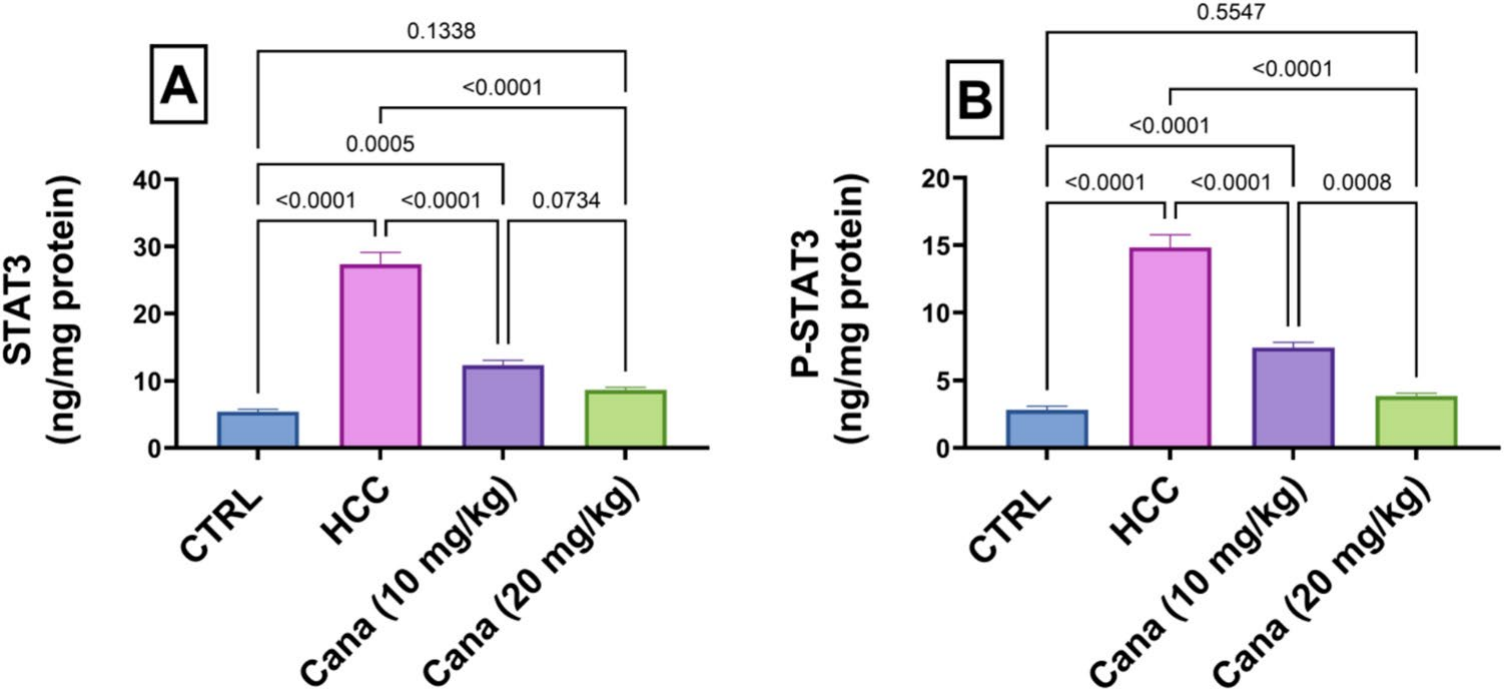
Assessment of canagliflozin effect on liver STAT3 and p-STAT3 in HCC rats. **A** STAT3 (ng/mg protein). **B** p-STAT3 (ng/mg protein). Data are presented as mean ± SEM. The exact *p*-value between each compared pairwise group is stated above the pairwise bar. HCC, hepatocellular carcinoma; Cana, canagliflozin

Liver STAT3 levels in the HCC group increased to 506.1% of the control group (*p* ≤ 0.05). Treatment with Cana at 10 mg/kg and 20 mg/kg reduced STAT3 levels to 45.2% and 31.6% of the HCC group, respectively (*p* ≤ 0.05). P-STAT3 levels in the HCC group increased to 526.4% of the control group (*p* ≤ 0.05). Treatment with Cana at 10 mg/kg and 20 mg/kg reduced P-STAT3 levels to 49.9% and 25.8% of the HCC group, respectively (*p* ≤ 0.05). The P-STAT3/STAT3 ratio in the HCC group was 1.02, compared to 0.52 in the control group. Treatment with Cana at 10 mg/kg and 20 mg/kg restored the ratio to 0.55 and 0.54, respectively, indicating normalization of inflammatory signaling (*p* ≤ 0.05) (Fig. 8).

### Effects of canagliflozin on HIF-1α mRNA expression and HIF-1α protein in HCC

**Fig. 9:**
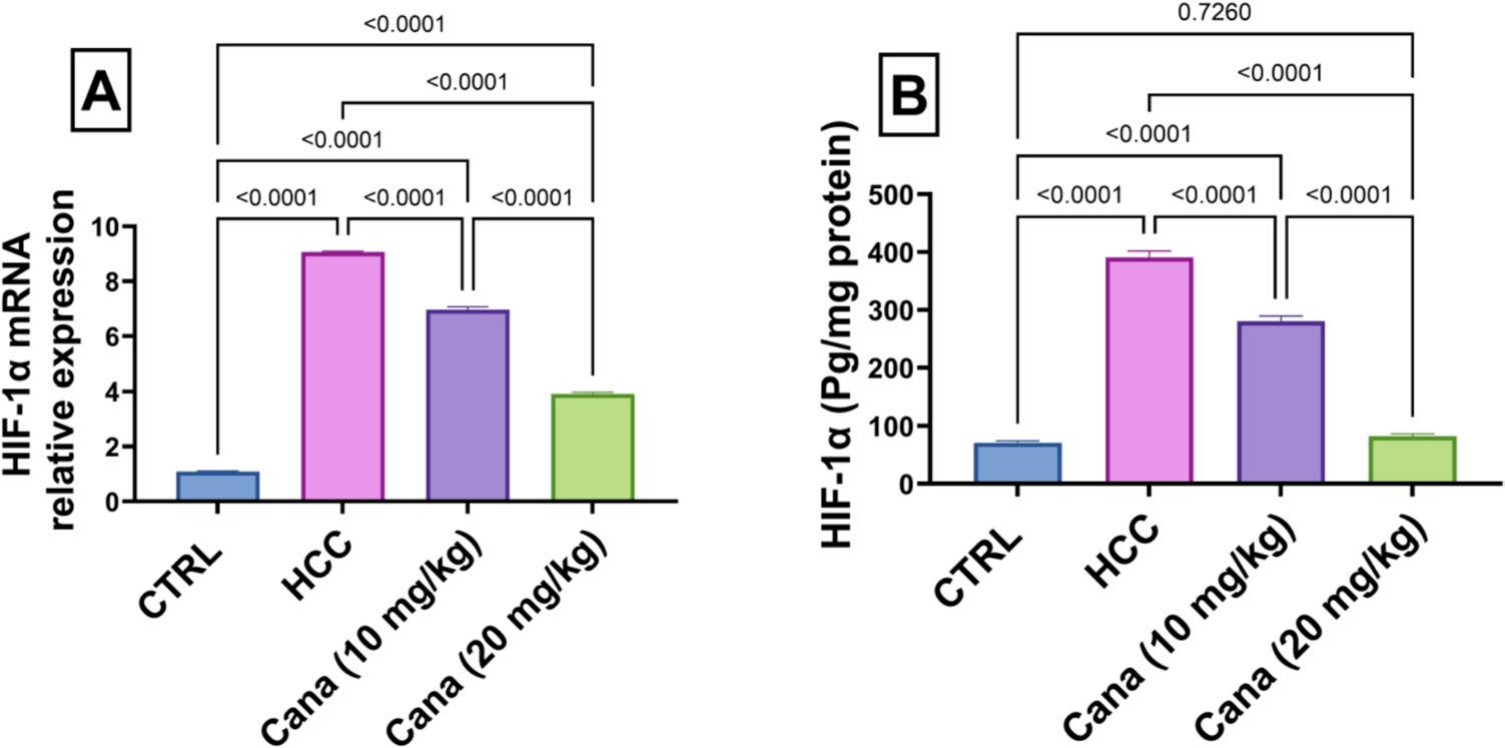
Assessment of canagliflozin effect on liver HIF-1α and HIF-1α gene expressions in HCC rats. **A** HIF-1α. **B** HIF-1α gene expressions. Data are presented as mean ± SEM. The exact *p*-value between each compared pairwise group is stated above the pairwise bar. HCC, hepatocellular carcinoma; Cana, canagliflozin

Liver HIF-1α protein levels in the HCC group increased to 549.2% of the control group (*p* ≤ 0.05). Treatment with Cana at 10 mg/kg and 20 mg/kg reduced HIF-1α levels to 71.7% and 21.1% of the HCC group, respectively (*p* ≤ 0.05). Similarly, HIF-1α mRNA expression in the HCC group increased to 831.7% of the control group (*p* ≤ 0.05). Treatment with Cana at 10 mg/kg and 20 mg/kg reduced HIF-1α mRNA expression to 76.8% and 43.2% of the HCC group, respectively (*p* ≤ 0.05) (Fig. 9).

### Gross liver findings

**Fig. 10:**
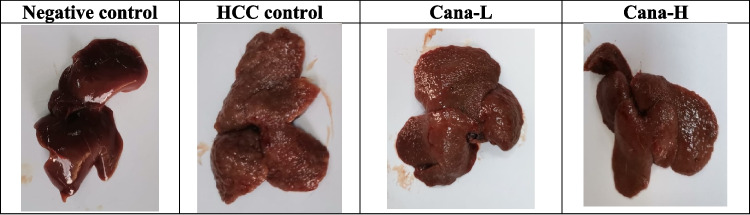
Macroscopic appearance of the liver of rats

During necropsy, each rat group’s liver was examined for any obvious abnormalities. It was revealed that the liver of the control group showed a normal appearance. Contrarily, the liver of the HCC-induced control group was pale, exhibiting irregularities and nodules, with the presence of lumps inside the liver tissue. The liver of animals treated with CANA revealed rough fibrotic surface with more improvement especially in the high-dose group (Fig. 10).

### Results of histopathology

**Fig. 11:**
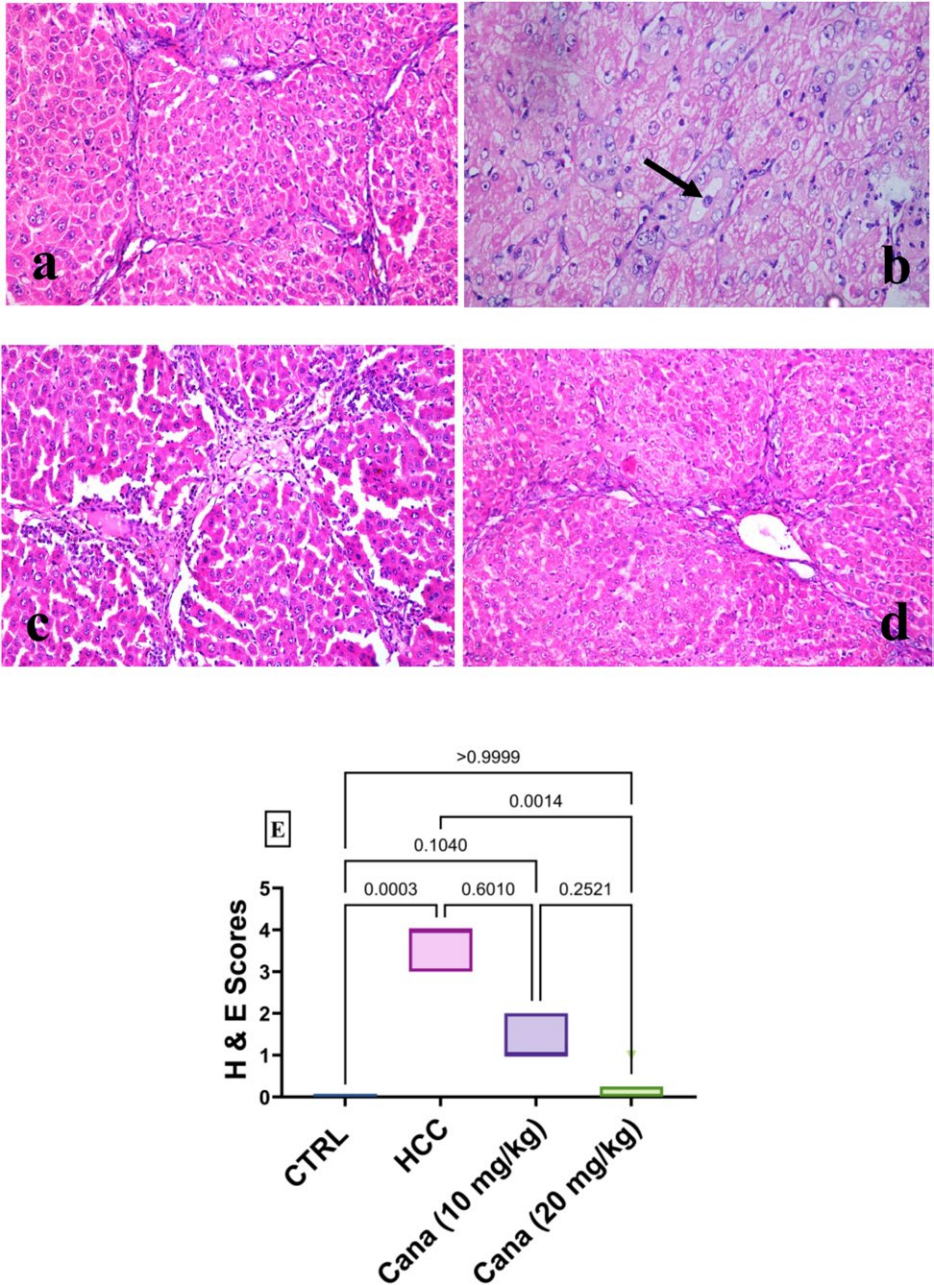
**a**–**d** Hepatocellular photomicrographs from several experimental groups stained with H&E X200 reveal: “**a** Normal group (0), **b** HCC group with HCC lesions in the form of acinus-like structure (arrow) (+ + +) among all hepatic lobules, **c** CANA (10 mg/kg) group with bridging fibrosis and formation of cirrhotic nodules (+), and **d** CANA (20 mg/kg) group with normal hepatic lobules with no interlobular reaction (0).” **E** Box and whiskers plot represents the median ± interquartile range and analyzed by the Kruskal–Wallis test followed by Dunn’s test. The exact *p*-value between each compared pairwise group is stated above the pairwise bar. HCC, hepatocellular carcinoma; Cana, canagliflozin

The normal group’s liver displayed typical hepatic parenchyma, including normal portal regions, blood sinusoids, hepatocytes, and central veins (Fig. 11a), while the liver from the HCC group revealed HCC lesions in the form of acinus-like structure with minimal atypia among all hepatic lobules, together with severe interlobular reaction in the form of congestion, fibrosis, and leucocytic cell infiltrations (Fig. 11b). Livers from the CANA (10 mg/kg) group showed the absence of HCC lesions with mild interlobular reaction (Fig. 11c), while the liver from the CANA (20 mg/kg) group revealed the absence of HCC lesions with no interlobular reaction (Fig. 11d). Histopathological grading of lesions in liver sections is listed in Table 2.

**Table 2:** Grading of histopathological lesions in the liver

Groups	Lesion grades			
	0 (negative)	+ (mild)	+ + (moderate)	+ + + + (severe)
CTRL	√			
HCC				√
CANA (10 mg/kg)		√		
CANA (20 mg/kg)	√			

### Histopathological lesion scoring

#### Immunohistochemical staining

**Fig. 12:**
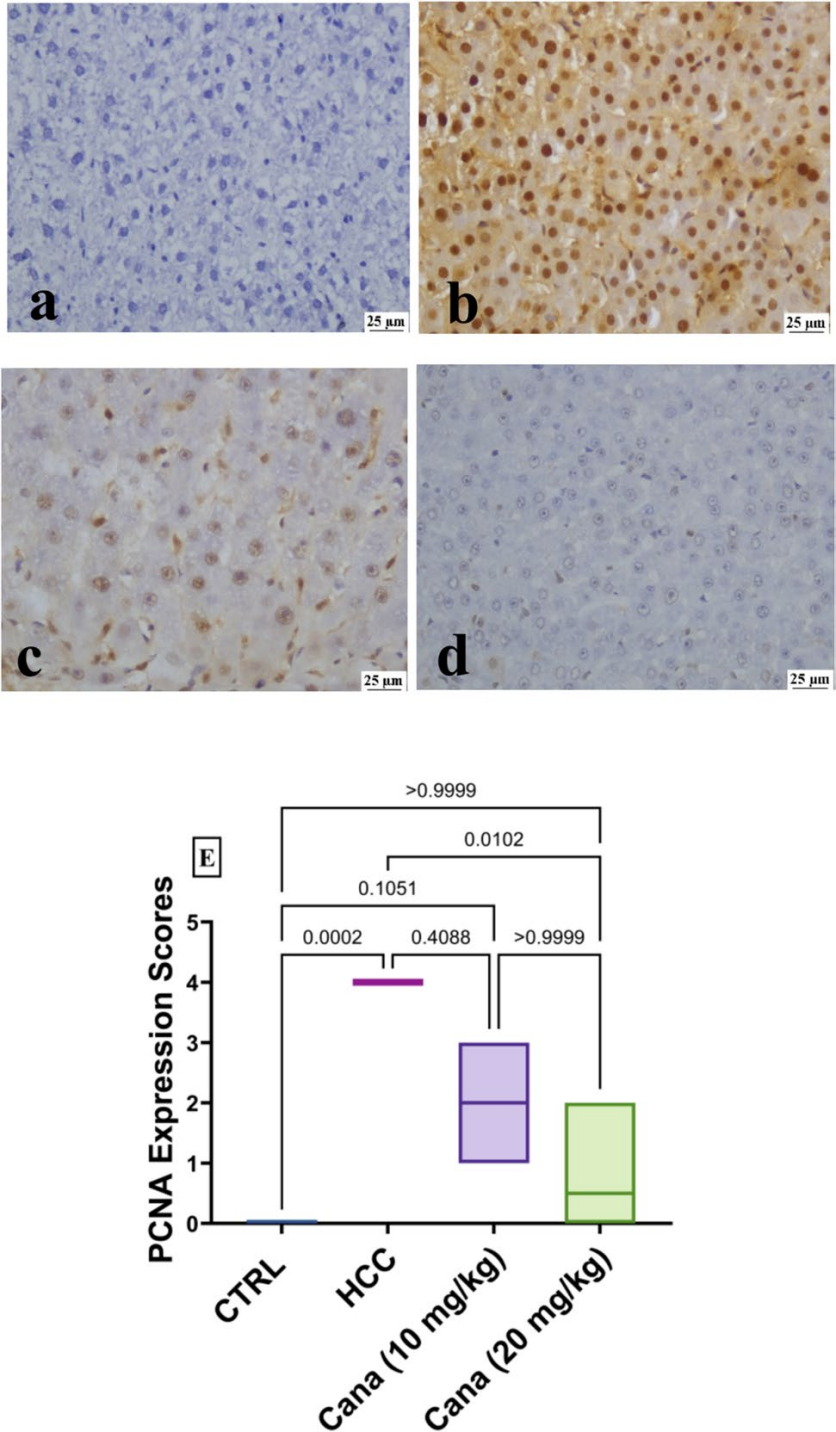
**a**–**d** Expression of proliferating cell nuclear antigen (PCNA) in liver tissues from different experimental groups showing the nucleus’s PCNA-positive cell staining was brown-yellow and appeared finely granular. **a** Normal group (0), **b** HCC group (+ + +), **c** CANA (10 mg/kg) group (+), and **d** CANA (20 mg/kg) group (0). **E** Box and whiskers plot represents the median ± interquartile range and analyzed by the Kruskal–Wallis test followed by Dunn’s test. The exact *p*-value between each compared pairwise group is stated above the pairwise bar. HCC, hepatocellular carcinoma; Cana, canagliflozin

**Fig. 13:**
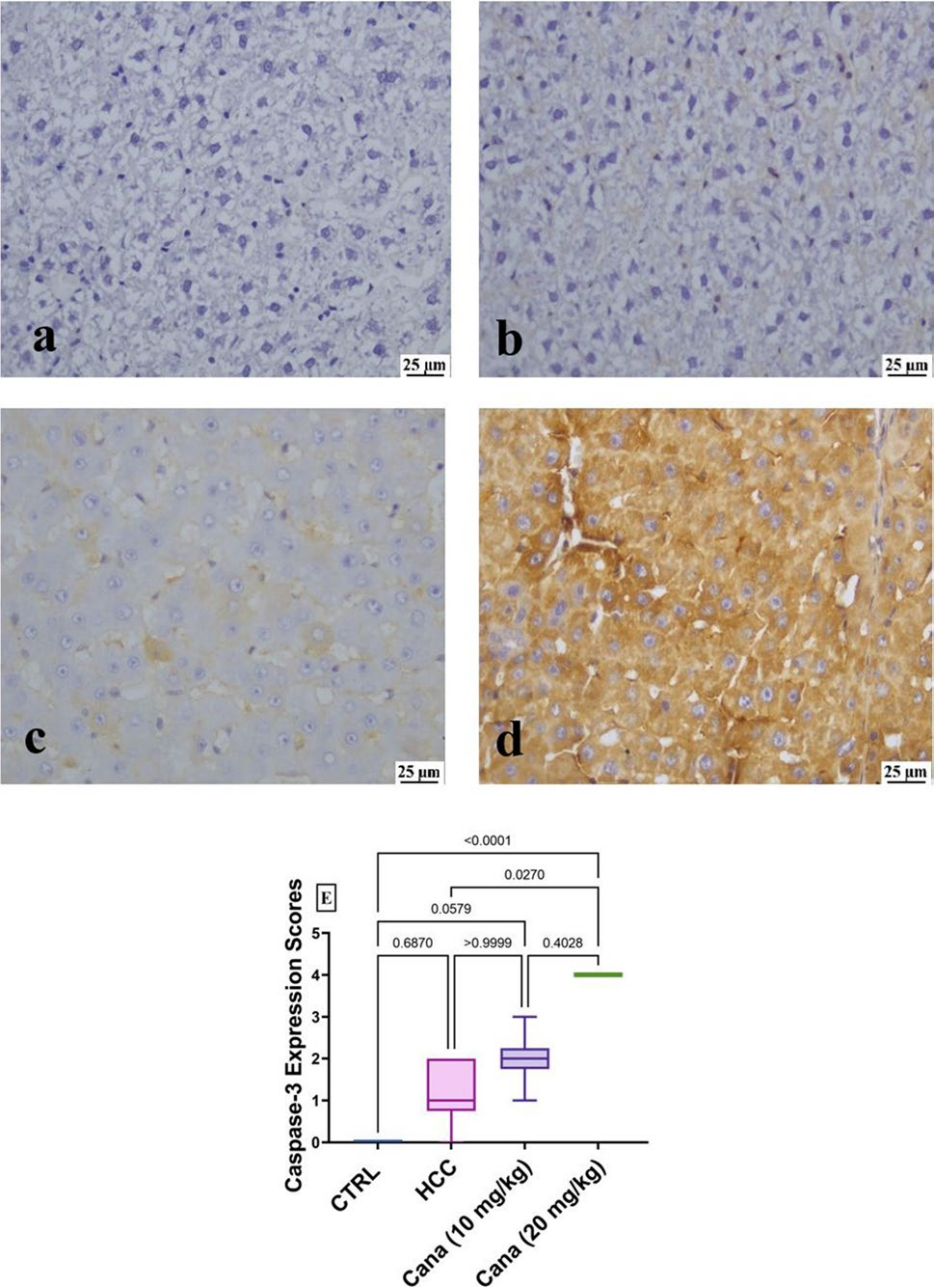
Expression of caspase-3 in liver tissues from different experimental groups showing: the brown granules that were Caspase-3 positive cells were primarily expressed in the portal region surrounding the hepatocytes’ cytoplasm, with minimal expression in the nucleus. **a**. Normalgroup (0), **b**. HCC group (0), **c**. CANA (10 mg/kg) group (+), **d**. CANA (20 mg/kg) group (++). **E**. Box and Whiskers plot represents median ± interquartile range and analyzed by Kruskal–Wallis test followed by Dunn’s test. The exact *p*-value between each compared pairwise group is stated above the pairwise bar. HCC: Hepatocellular carcinoma; Cana: Canagliflozin

By immunohistochemistry, staining of the CANA (10 mg/kg) group, and the HCC group tissues using PCNA-specific monoclonal antibody, the expression of PCNA was found. The nucleus’s PCNA-positive cell staining was brown-yellow and appeared finely granular. PCNA expression was strong in the control positive group and mild in CANA (10 mg/kg) (Fig. 12a–d). The brown granules that were caspase-3-positive cells were primarily seen in the portal region surrounding the hepatocytes’ cytoplasm; however, there were also some nuclear expressions. Caspase-3 expression was mild in the HCC group and strong in the CANA (10 mg/kg) group (Fig. 13a–d).

## Discussion

Numerous studies have looked into developing HCC animal models to lower “the time-to-death ratio” and restrict the carcinogen doses administered. Thus, we employed a two-stage paradigm in the current investigation that involved a single dosage of a genotoxic diethylnitrosamine (DEN) for initiation and the non-genotoxic compound thioacetamide (TAA) as a cancer promoter, following a choline-deficient diet (CDD) (Kimura et al. 2013). The gene expression patterns of chemically derived HCC models are similar to the poor prognosis linked to human HCC (Singh et al. 2018; Rahman et al. 2019). The CDD was employed in our study’s hepatocarcinogenesis model. Deficit of choline causes lipid peroxidation, mitochondrial malfunction, overexpression of cytochrome P_450_ 2E1 (CYP2E1), and increased generation of reactive oxygen species (ROS) (Lima et al. 2008). When cytochrome P_450_ bioactivates DEN, mutagenic DNA adducts are formed. Additionally, reactive oxygen species (ROS) are formed, launching oxidation stress, DNA damage, and, ultimately, the promotion of hepatocarcinogenesis (Valko et al. 2006). Additionally, cytochrome P_450_ enzymes bioactivate TAA to become TA sulfoxide (TAASO) and then TAASO2, which are hazardous substances that increase ROS formation, lipid peroxidation, cytotoxicity, and mitochondrial damage. Eventually, this results in apoptosis, centrilobular necrosis, and the development of HCC (Rekha et al. 2008; Ramadan et al. 2018). Consequently, the current study’s goal is to determine whether CANA may treat CDD/DEN/TAA-induced HCC in a rat animal model.

Current investigation revealed a substantial increase in the serum GGT, AST, and ALT levels in the HCC group, which highlighted the extent of hepatocyte necrosis (Abdel-Rahman et al. 2022; Nyblom et al. 2004). This increase could be the result of hepatic necrosis and the consequent release of these enzymes from cancerous cells or irreparably damaged hepatic cells into circulation (Abdo et al. 2015). Treatment with CANA, especially at a dose of 20 mg/kg, normalized the aberrant serum levels. Canagliflozin normalized liver enzyme activity which was corroborated by Wang’s study (Wang et al. 2021).

Furthermore, the transformation of liver injury to HCC in our investigation was demonstrated by increased tumor markers (AFP, AFU, CEA, TGF-β1, YAP, TAZ, and HIF-1α) and a histological analysis of the liver that showed an increase in the size and quantity of tumor nodules in the HCC group. Alpha-fetoprotein (AFP) is frequently used for HCC diagnosis, screening, and treatment monitoring (Sauzay et al. 2016). Hepatocytes showed evidence of AFP gene reactivation in the early stages of hepatocarcinogenesis; cytoplasmic AFP promotes the growth of cancerous liver cells (Lu et al. 2016). According to the current study, the HCC group showed an increase in serum AFP, which aligns with those of Zhang et al. (2016) who showed elevated levels of AFP in HCC rats. Furthermore, the present study showed that the HCC group had marked elevated levels of alpha-l-fucosidase (AFU), which is consistent with previous findings (Abdallah and Khattab 2004). AFU is a significant HCC biomarker since it is raised in HCC patients (Mohamed et al. 2019). Additionally, Moriwaki et al. (2007) have revealed that there is an increase in AFU enzyme levels as a result of the fucosylation of sugar proteins during the course of hepatocarcinogenesis. Additionally, carcinoembryonic antigen (CEA) was assayed in our study, which is used for the clinical diagnosis of gastrointestinal cancer (Yang et al. 2018); besides, certain patients with a poor prognosis for HCC have a higher CEA level (Yoshikawa et al. 2017). CANA-treated groups demonstrated a significant decrease in AFP, AFU, and CEA levels. In line with our findings, a mouse model of diabetes and non-alcoholic steatohepatitis-related hepatocarcinogenesis, Jojima et al. (Jojima et al. 2019) showed that CANA prevents carcinogenesis. All of these results point to the effectiveness of canagliflozin treatment for anti-hepatocarcinogenesis, as it decreased the levels of tumor markers: AFP, AFU, and CEA.

Solid tumors, such as HCC, frequently experience oxygen deprivation, which stimulates hypoxia-inducible factor-1 alpha (HIF-1α) to increase angiogenesis and the creation of vascular endothelial growth factor (VEGF) (Carbajo-Pescador et al. 2013). Furthermore, the transforming growth factor β1 (TGF-β1) level is highly expressed in cancerous cells during hypoxia through HIF-1α, according to Deng (Deng et al. 2013). The HCC group in the present study showed an increase in HIF-1α and TGF-β1 levels. Tamim et al.’s study (Tamim et al. 2024) found that the HCC group caused by DEN had higher levels of TGF-β1 and HIF-1α, which is consistent with these findings. Additionally, the Hippo pathway’s major downstream terminal effectors are YAP and its homolog transcriptional co-activator TAZ (Lei et al. 2008). In malignancies, YAP/TAZ are moved to the nucleus and attach to TEAD (TEA domain) proteins, which leads to the expansion of organ size through cell proliferation, apoptosis prevention, and progenitor/stem cell amplification (Cai et al. 2010; Hong and Guan 2012). Overexpression of the YAP/TAZ genes has been found in liver cancer patients (Pan 2010). Interestingly, treatment with CANA led to a significant drop in hypoxia and fibrosis markers levels, which included AFP, AFU, and TGF-β1.

Furthermore, AMPK, “a cellular energy stress sensor” that is triggered by elevated AMP levels, has the ability to regulate cellular metabolism and work in tandem with available energy to coordinate cell development. Active AMPK is lower in the tumor compared to the surrounding tissue in patients with liver cancer (Lee et al. 2012). Inhibition of YAP by AMPK has been documented (Zhao et al. 2007). AMPK is essential for YAP suppression because it mediates YAP phosphorylation, specifically on Ser 94, and prevents the formation of the YAP-TEAD complex (Li et al. 2010). Current findings revealed that CANA increased energy metabolism markers: AMPK, P-AMPK, AMPK mRNA relative expression, and P-AMPK/AMPK ratio.

SGLT2 is overexpressed in many kinds of cancer, providing the glucose that cancer cells need to meet their high energy requirements (Scafoglio et al. 2015). One of the potential molecular mechanisms is that it prevents the renal tubules from re-absorbing glucose, which lowers the amount of glucose needed for tumor cell development and metabolism (Dutka et al. 2022), so preventing tumor cell growth and proliferation. SGLT2 suppression consistently inhibited the growth of tumors in mice (Nasiri et al. 2019). There is an increasing evidence that SGLT2 inhibition may reduce the growth of carcinomas that express SGLT2, including breast (Zhou et al. 2020), cervical (Xie et al. 2020), hepatocellular (Shiba et al. 2018), prostate, and lung carcinomas (Villani et al. 2016). According to Kaji et al., by inhibiting the uptake of glucose, the formation of lactate, and the synthesis of intracellular ATP, canagliflozin prevents the growth of HepG2 cells (Kaji et al. 2018). AMPK activation and decreased ATP synthesis result from the inhibition of mitochondrial complex I by canagliflozin, a strong SGLT2 inhibitor (Hawley et al. 2016). Inhibiting mTOR through AMPK activation results in cell cycle arrest and the production of apoptosis (Motoshima et al. 2006). By suppressing forkhead box A1 and sonic hedgehog signaling, activated AMPK prevents the growth of tumor cells and triggers apoptosis (Xie et al. 2020). Canagliflozin, through AMPK activation and mTOR inhibition, downregulates HIF-1α, hence inhibiting the proliferation of non-small cell lung cancer (NSCLC) and improving the efficacy of radiation therapy (Biziotis et al. 2023).

In line with previous studies, CANA therapy, particularly high doses, showed a marked decrease in HIF-1α, TGF-β1, and YAP/TAZ levels and exhibited a marked increase in AMPK and p-AMPK levels and expression. The idea that CANA may also inhibit intra-tumor neovascularization supports our findings (Polet and Feron 2013). These findings, with the previous findings related to AFP, CEA, and AFU, confirmed that CANA treatment has anti-tumor and anti-angiogenic effects.

Further, canagliflozin suppressed oxidative stress and enhanced the levels of antioxidant markers: MDA, SOD, and GSH, evidenced by measuring lipid peroxide as MDA is one of the most prominent indicators of oxidation stress (Elbaset et al. 2023). Our findings showed a notable increase in MDA and a large decrease in GSH and SOD, the antioxidants essential for MDA scavenging. The decline in the cellular antioxidants is indicative of the oxidative toxic impact of DEN/TAA. This result was corroborated by Zhang et al.’s study (Zhang et al. 2016) which discovered that DEN can stimulate HCC progression via interacting with macromolecules such as DNA, lipids, anti-oxidative enzymes, and enzymes involved in the DNA repair pathway. Furthermore, TAA biotransformation produces extremely reactive compounds that bind covalently to liver macromolecules, initiating hepatic damage and inhibiting SOD activities and GSH content (Elbaset et al. 2024). HCC rats exhibited notably reduced hepatic MDA levels and increased GSH content and SOD activity following CANA administration. The findings of this study may indicate that CANA can lessen hepatic oxidative damage and, in turn, lessen HCC progression. The antioxidant properties of CANA have been proven in multiple studies using experimental disease models (Kabil and Mahmoud 2018; Hasan et al. 2020).

In 55% of HCC patients, sirtuin 1 (SIRT1) is overexpressed, which is linked to a greater tumor grade and a lower chance of survival. Due to SIRT1’s ability to modify the activity of proteins essential for carcinogenesis, it promotes both drug resistance and tumor growth and progression (Chen et al. 2012; Ling et al. 2017). The deacetylase SIRT1 is dependent on nicotinamide adenine dinucleotide (NAD). Because cancer cells undergo metabolic alterations, they need extra NAD (as a redox partner) in cellular energy metabolism processes. Important biological functions required for the proliferation of cancer cells, such as “transcription, cell-cycle progression, anti-apoptosis, and DNA repair,” are regulated by NAD-dependent enzymes like SIRT1 (Garten et al. 2015; Revollo et al. 2004). According to our data, the HCC group’s SIRT1 levels significantly increased. These results were consistent with the Chen et al. study (Chen et al. 2020), which discovered that SIRT1 was substantially more in HCC tissues compared to nearby normal tissues. Meanwhile, treatment with canagliflozin restored the elevated Sirt1 to normal levels.

Nrf2 regulates and encodes antioxidant proteins through its interaction with the antioxidant-responsive element (ARE) (Elbaset et al. 2024). Nrf2 normally binds with “the Kelch-like ECH-associated protein-1 (Keap1)” in the cytoplasm (Raslan et al. 2021). Upon exposure to DEN/TAA free radicals, Nrf2 is liberated from Keap1 and moves into the nucleus, triggering the antioxidant genes (Rotblat et al. 2012; Abdel-Rahman et al. 2024). It is significant that prior research has demonstrated the crucial function of the NRF2/KEAP1 signaling pathway in carcinogenesis and the relationship between redox and metabolism in cancer (Hayes and Dinkova-Kostova 2014), providing additional evidence that Nrf2 acts as an oncogene to encourage tumor cell motility and invasion, potentially by boosting the tumor’s resistance to oxidative stress. It is increasingly evident that the NRF2/KEAP1 pathway contributes significantly to the metabolic reprogramming of cancer cells by means of a transcriptional program that promotes cancer cell proliferation and malignant development. Furthermore, Nrf2 promotes intermediate metabolism by means of glutaminolysis (Romero et al. 2017), which leads to an imbalance in metabolic activities, including nucleotide (Mitsuishi et al. 2012) and amino acid biosynthesis (DeNicola et al. 2015). Our findings showed that Nrf2 plays an oncogenic role in the formation of liver cancer since the HCC group had elevated Nrf2 levels, which were reversed to normal levels in the groups receiving CANA treatment.

Signal transducer and activator of transcription 3 (STAT3) is a versatile transcription factor that is intimately linked to the transformation, survival, invasion, proliferation, and metastasis of tumor cells as well as the tumor inflammatory response (Tolomeo and Cascio 2021). Numerous cytokines, growth factors, hormones, and hepatitis virus proteins all contribute to the hepatic activation of STAT3. Among these, IL-6 and IL-22 are two strong inducers of hepatocyte STAT3 activation (Wang et al. 2011). The hyperactivity of STAT3 in HCC cells has been demonstrated to enhance the malignant biological behavior of tumor cells by promoting their invasion, metastasis, and proliferation while also preventing their apoptosis (Yuen et al. 2010). According to Li et al. (2006) RNA interference on STAT3 dramatically decreased tumor volume and suppressed cell proliferation in mice. Furthermore, STAT3 activation can trigger the production of numerous downstream genes that impact the pathogenesis of various malignancies, including Bcl-2 (Levy and Darnell 2002). Canagliflozin treatment significantly decreased STAT3 and p-STAT3 levels, which increased markedly in the HCC group. According to a prior study, conditional deletion of STAT3 in hepatocytes stops the formation of liver tumors caused by a single injection of DEN (He et al. 2010) which confirmed our findings.

Canagliflozin may mitigate the hyperinsulinemic state and diminish the related risk factors for liver carcinogenesis by lowering blood glucose levels and enhancing insulin sensitivity. Decreased insulin transmission may consequently diminish the proliferative signals that promote cancer in the liver. Canagliflozin has demonstrated the capacity to diminish systemic inflammation, maybe via its influence on metabolic regulation. The medicine may diminish inflammatory cytokine cascades (e.g., TNF-α, IL-6) by reducing glucose levels and enhancing insulin sensitivity, so mitigating liver damage and fibrosis, which elevate the risk of liver cancer. Furthermore, enhanced glucose metabolism may diminish the liver’s exposure to advanced glycation end-products (AGEs), which are byproducts of non-enzymatic glucose metabolism that provoke inflammation and tissue damage. Canagliflozin’s capacity to decrease blood glucose levels may diminish the production of reactive oxygen species (ROS) and oxidative injury to hepatic cells, therefore alleviating a principal mechanism that fosters carcinogenesis (DeFronzo et al. 2015; Wanner et al. 2016; Mohamed et al. 2016; Davidson 2019).

Both an increase in cell proliferation and a decrease in cell death can lead to the growth of tumors. Dysregulation of apoptosis (programmed cell death) is believed to contribute to cancer progression (Wyllie 1997). Apoptotic cell death is initiated and carried out by members of the caspase family of cellular proteases. An important protease in the apoptotic process is caspase-3 (McIlwain et al. 2013). Immune expression of the cell proliferation marker, proliferating cell nuclear antigen (PCNA), and caspase-3 showed that the HCC group had significantly higher expression of PCNA and significantly lower expression of caspase-3. After CANA therapy, PCNA expression decreased, and caspase-3 expression increased. Comparable results were reported in a study by Jojima et al. (Jojima et al. 2019) that showed canagliflozin activated caspase-3 to induce apoptosis in HepG2 cells.

In rats, SGLT2 inhibitors like canagliflozin have demonstrated advantageous benefits in diminishing hepatic steatosis, inflammation, and fibrosis. These are all essential antecedents to the progression of HCC. Given that metabolic disorders such as insulin resistance, hyperglycemia, and non-alcoholic fatty liver disease (NAFLD) are pivotal to the advancement of human HCC, the results reported in rats (e.g., enhanced glucose metabolism and diminished liver fat) may be pertinent for human therapeutic applications. Nonetheless, the specific metabolic pathways may vary marginally among species, and the severity of HCC in humans is affected by various factors, including genetic predisposition, viral infections (such as hepatitis B or C), and environmental influences, which may not be entirely mirrored in rat models (Xu et al. 2022).

Rats treated with SGLT2 inhibitors have demonstrated reduced liver inflammation and fibrosis, which are critical components in the progression from liver disease to HCC. These findings are relevant to humans, as chronic inflammation and liver fibrosis are major risk factors for HCC. In humans, inflammation from conditions like NAFLD and NASH (non-alcoholic steatohepatitis) plays a key role in driving tumorigenesis (Shiba et al. 2018).

SGLT2 inhibitors, such as canagliflozin, have demonstrated a reduction in oxidative stress in animal models, a critical element in hepatic injury and carcinogenesis. Given that oxidative stress has a role in DNA damage and cancer in both rats and humans, this phenomenon is very pertinent. Mitigating oxidative stress may potentially decelerate the advancement of liver disease and diminish the probability of HCC occurrence in humans. Although oxidative stress significantly impacts both rats and people, the regulatory mechanisms of oxidative damage may vary, necessitating long-term investigations in humans to validate these effects (Davidson 2019).

The results from rat models offer a promising basis for investigating the possible application of SGLT2 inhibitors, such as canagliflozin, in halting the advancement of liver disease and mitigating the risk of HCC. The decrease in hepatic fat, inflammation, fibrosis, and oxidative stress in rats are mechanisms that are significantly pertinent to human liver disease. Nonetheless, despite the encouraging benefits, the translation of findings from animal models to human treatment necessitates additional clinical research to validate their efficacy and safety in human populations. Future research must meticulously evaluate human-specific aspects, including genetic variations and the multifactorial characteristics of HCC. Human clinical trials and longitudinal investigations are necessary to verify the efficacy of SGLT2 inhibitors in mitigating HCC risk or enhancing outcomes in individuals with liver disease.

## Conclusion

The current study showed that canagliflozin effectively lessens liver injuries and oxidative stress, which occur in HCC in rats after exposure to CDD, DEN, and TAA. The canagliflozin doses of 10 and 20 mg/kg were observed to have considerably reduced the elevated liver enzymes of ALT, AST, and GGT, along with an improvement in liver function. Correspondingly, oxidative stress markers-MDA were considerably decreased, accompanied by elevated levels of antioxidant markers such as SOD and GSH, which significantly improved upon the possible damage caused by oxidative stress.

In addition, treatment with canagliflozin led to a significant drop in tumor marker levels, which included AFP, AFU, and CEA, potentially influencing the tumor during development. Also, a modulation was demonstrated in significant pathways, such as HIF-1α or YAP1, TAZ. Expression of TGF-β1, a discovery related to the progression and development of cancer, is in altered expression.

Moreover, canagliflozin showed strong beneficial effects on energy demand and inflammatory response, evidenced by AMPK and STAT3 signal transduction pathways. The excellent improvements in biochemical and histopathological parameters point to the therapeutic potential of canagliflozin with respect to liver injury and HCC. Eventually, the notable enhancements in biochemical and histological measurements demonstrated the therapeutic potential of canagliflozin for liver damage and HCC.

## Data Availability

All source data for this work (or generated in this study) are available upon reasonable request.
